# Endocrine disruptors in plastics alter β-cell physiology and increase the risk of diabetes mellitus

**DOI:** 10.1152/ajpendo.00068.2023

**Published:** 2023-05-03

**Authors:** Juan Martínez-Pinna, Roberto Sempere-Navarro, Regla M. Medina-Gali, Esther Fuentes, Ivan Quesada, Robert M. Sargis, Leonardo Trasande, Angel Nadal

**Affiliations:** ^1^Instituto de Investigación, Desarrollo e Innovación en Biotecnología Sanitaria de Elche (IDiBE), Universidad Miguel Hernández de Elche, Elche, Spain; ^2^Departamento de Fisiología, Genética y Microbiología, Universidad de Alicante, Alicante, Spain; ^3^CIBER de Diabetes y Enfermedades Metabólicas Asociadas (CIBERDEM), Instituto de Salud Carlos III, Madrid, Spain; ^4^Division of Endocrinology, Diabetes, and Metabolism, Department of Medicine, University of Illinois at Chicago, Chicago, Illinois, United States; ^5^Department of Pediatrics, New York University Grossman School of Medicine, New York, New York, United States; ^6^Department of Population Health, New York University Grossman School of Medicine, New York, New York, United States; ^7^Wagner School of Public Service, New York University, New York, New York, United States

**Keywords:** β-cells, diabetes, endocrine disruptors, insulin resistance, insulin secretion

## Abstract

Plastic pollution breaks a planetary boundary threatening wildlife and humans through its physical and chemical effects. Of the latter, the release of endocrine disrupting chemicals (EDCs) has consequences on the prevalence of human diseases related to the endocrine system. Bisphenols (BPs) and phthalates are two groups of EDCs commonly found in plastics that migrate into the environment and make low-dose human exposure ubiquitous. Here we review epidemiological, animal, and cellular studies linking exposure to BPs and phthalates to altered glucose regulation, with emphasis on the role of pancreatic β-cells. Epidemiological studies indicate that exposure to BPs and phthalates is associated with diabetes mellitus. Studies in animal models indicate that treatment with doses within the range of human exposure decreases insulin sensitivity and glucose tolerance, induces dyslipidemia, and modifies functional β-cell mass and serum levels of insulin, leptin, and adiponectin. These studies reveal that disruption of β-cell physiology by EDCs plays a key role in impairing glucose homeostasis by altering the mechanisms used by β-cells to adapt to metabolic stress such as chronic nutrient excess. Studies at the cellular level demonstrate that BPs and phthalates modify the same biochemical pathways involved in adaptation to chronic excess fuel. These include changes in insulin biosynthesis and secretion, electrical activity, expression of key genes, and mitochondrial function. The data summarized here indicate that BPs and phthalates are important risk factors for diabetes mellitus and support a global effort to decrease plastic pollution and human exposure to EDCs.

## Graphical abstract

**Figure:**
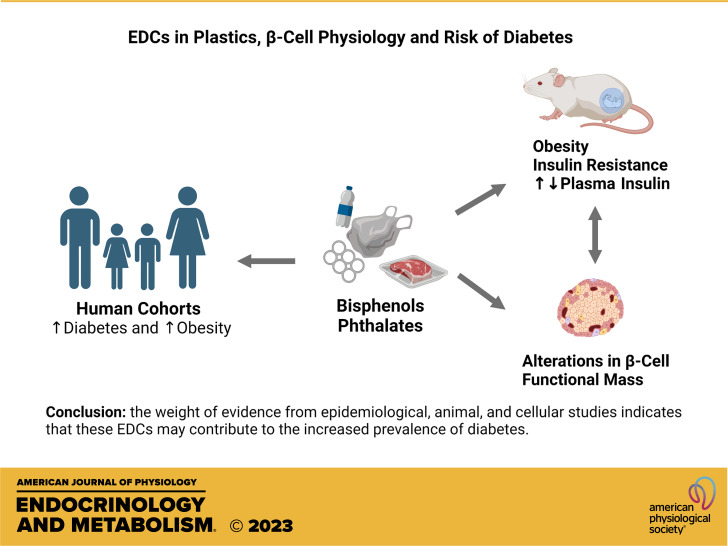

## INTRODUCTION

Plasma glucose levels must be maintained within the normoglycemic range of 80–110 mg/dL (4.4–6.1 mM) to preserve proper bodily function. Sustained glucose levels above 7.2 mM are diagnosed as diabetes mellitus, defined by the American Diabetes Association as “a group of metabolic diseases characterized by hyperglycemia resulting from defects in insulin secretion, insulin action, or both” (1). Traditionally, the disorder has been classified as type 1 diabetes (T1D), type 2 diabetes (T2D), and other less common forms such as gestational diabetes or monogenic diabetes. The most common form is T2D whose etiology includes both genetic susceptibility (2, 3) and environmental factors, including exposure to environmental pollutants (4–6).

Obesity is the main risk factor for T2D. Although not all individuals with obesity develop metabolic problems, many exhibit hyperinsulinemia and insulin resistance, two conditions that promote T2D pathogenesis (2, 7). Hyperinsulinemia is primarily caused by increased insulin synthesis and release from pancreatic β-cells, either as a prior or compensatory response to insulin resistance (7). Individuals in whom functional β-cell mass declines over time will show impaired glucose tolerance leading to T2D. This loss of functional β-cell mass occurs due to failure of stimulus-secretion coupling and/or cell death or dedifferentiation.

Insulin sensitivity and insulin secretion are interrelated variables exhibiting a hyperbolic relationship (8), which explains why insulin-resistant subjects secrete 2–5 times more insulin in response to glucose, whereas highly insulin-sensitive athletes secrete 2–5 times less insulin (9, 10). During critical periods of development such as puberty and pregnancy, functional β-cell mass increases while insulin sensitivity decreases, effects mediated by sex and maternal hormones (11–14). Thus, insulin sensitivity and β-cell function are interrelated variables. Insulin-resistant subjects, whether obese or lean, have higher insulin secretion and lower insulin clearance than insulin-sensitive individuals.

**Figure 1.**
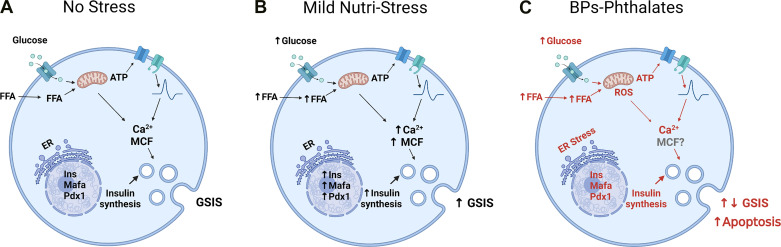
Bisphenols (BPs) and phthalates interfere with the biochemical basis of β-cell adaptation to mild nutri-stress. *A*: under physiological circumstances, in the absence of metabolic stress, pancreatic β-cells respond to an increase in extracellular glucose or other nutrients such as free fatty acid (FFA) with an increase in ATP due to the metabolism of these nutrients and the production of metabolic coupling factors (MCF), initiating the stimulus-secretion coupling process as well as increasing insulin biosynthesis. *B*: mild nutri-stress occurs during overweight/obesity and increase pancreatic functional mass. This consist of an increase in MCF, insulin synthesis, regulation of electrical activity, elevated basal and stimulated insulin release and gene expression of β-cell markers (22). This condition prevents the debut of diabetes but courses with hyperinsulinemia, insulin resistance, and mild hyperglycemia. When nutri-stress becomes more severe, β-cell collapses and diabetes starts. *C*: bisphenols (BPs) and phthalates target the same biochemical pathways activated by mild nutri-stress and therefore may disrupt the adaptation of β-cell to metabolic stressors such as overweight. They induce mitochondrial dysfunction and rise reactive oxygen species (ROS), increase or decrease insulin synthesis, depending on dose and duration of treatment, induce endoplasmic reticulum stress and decrease gene expression of β-cell markers. In the case of bisphenols, they decrease the expression and the activity of ion channels and modify glucose-induced electrical activity and Ca^2+^ signaling. The final result is an alteration of glucose-stimulated insulin secretion (GSIS) which is increased in the case of BPs and decreased in the case of phthalates, although there is variability of results depending on conditions. In general, there is increased β-cell apoptosis with both groups of endocrine disrupting chemicals (EDCs). Red color emphasizes the pathways changed by these EDCs in pancreatic β-cells. Created with BioRender.com.

Animal studies show that the β-cells adapt to obesity by increasing glucose-stimulated insulin secretion (GSIS) (8, 15) and β-cell mass (16, 17). Structural adaptations have been extensively studied and reviewed in both animals and humans (18–20). Murine β-cells also functionally adapt to insulin resistance resulting from high-fat diet-induced obesity. These functional changes include insulin hypersecretion that can maintain glucose homeostasis near that of control diet-fed mice (21). β-Cells from *ob/ob* obese mice, a genetic model of obesity, showed increased mitochondrial activity along with alterations in electrical activity, including increased frequency of action potentials and increased sensitivity to low glucose concentrations (15). These cells show more vigorous intracellular Ca^2+^ mobilization and increased insulin exocytosis. As a consequence of the upregulation of these processes, there is an increase in GSIS (Fig. 1, *A* and *B*).

These β-cell adaptations to obesity involve biochemical pathways triggered by higher levels of glucose, amino acids, and free fatty acids that increase glucose metabolism and mitochondrial lipid oxidation while increasing insulin synthesis and expression of transcription factors and β-cell differentiation markers, including MafA, Pdx1, NeuroD1, Nkx6.1, and Pax6 (22). Other signaling molecules from adipocytes (e.g., leptin, adiponectin) and proinflammatory cytokines from macrophages and other cells infiltrating adipose tissue [e.g., TNFα, IL-6 and monocyte chemoattractant protein (MCP-1)] also play a role (23, 24). β-Cell mass can increase via hypertrophy of existing cells and proliferation. Glucose, nonesterified fatty acids (NEFAs), incretins, and neuronal signaling all play a role in the regulation of β-cell functional mass (25). In conclusion, healthy β-cells adapt to compensate for insulin resistance and maintain normal glucose tolerance. However, if β-cells are dysfunctional due to genetic vulnerability, environmental factors, or both, then the individual may develop impaired glucose tolerance, elevated fasting glucose levels, and ultimately frank T2D.

Environmental factors that predispose β-cells to dysfunction include endocrine disrupting chemicals (EDCs). EDCs directly alter functional β-cell mass and decrease insulin secretion, accelerating the decline in functional β-cell mass and promoting T2D development (4, 6). Indirectly, EDCs can also act as obesogens, promoting the development of obesity and increasing insulin resistance, further augmenting T2D risk (26, 27). The following section will briefly describe what EDCs are, how they work, and which periods of life are most vulnerable.

## ENDOCRINE DISRUPTING CHEMICALS

### Overview of Endocrine Disrupting Chemicals

EDCs are defined by the International Programme on Chemical Safety/World Health Organization (IPCS/WHO) as “an exogenous substance or mixture that alters function(s) of the endocrine system and consequently causes adverse health effects in an intact organism, or its progeny, or (sub) populations” (World Health Organization, Global Assessment of the State-of-the-Science of Endocrine Disruptors, 2002) (28).

EDCs are a global problem with near-ubiquitous human exposure given their presence in a multitude of products that we use daily. Currently, more than 500 chemicals are listed as EDCs or suspected EDCs by the European Commission (https://ec.europa.eu/environment/chemicals/endocrine/strategy/substances_en.htm#priority_list). Some EDCs are classified as persistent organic pollutants (POPs) since they are stored in fat and remain in our bodies for long periods of time. POPs are released into the circulation during periods of fat mobilization, including during weight loss (29) as well as during pregnancy and lactation (30). Examples include dioxins, flame retardants, perfluorinated compounds, polychlorinated biphenyls (PCBs), and organochlorine, and organophosphate pesticides. Stored in animal fats, their concentration increases as they accumulate in the trophic chain, a process known as biomagnification.

Some POPs are diabetogenic because they directly modify β-cell function and/or mass (31). In contrast, other EDCs are nonpersistent, do not accumulate as readily, and are metabolized and excreted in ∼24 h; such EDCs include phthalates and most bisphenols. However, exposure to these EDCs is both high and frequent; as such, biomonitoring programs in the United States and Europe have detected them in the urine of most citizens (32).

EDCs act by modifying hormone function, disrupting homeostasis, and altering physiology throughout an individual's lifetime, from fetal development to adulthood (33, 34). Although EDCs vary in their mechanisms of hormonal disruption (e.g., altering hormone receptors expression or circulating hormone levels), many EDCs act by inappropriately binding to nuclear receptors (34, 35). The most studied nuclear receptors to which EDCs bind are the estrogen receptors (ER) and androgen receptors as well as the peroxisome proliferator-activated receptor γ (PPARγ), which is involved in the control of lipogenesis, and the aryl hydrocarbon receptor, which regulates expression of enzymes involved in the xenobiotic metabolism (34). They can also bind to the glucocorticoid receptor and the thyroid hormone receptor (34). These receptors act as transcription factors triggering nuclear-initiated actions that regulate gene transcription (36) or activate extranuclear-initiated actions that regulate a variety of signaling cascades (37). These receptors are promiscuous in that they bind multiple ligands in addition to their natural ligands; this includes binding EDCs, resulting in inappropriate gene expression and/or altered signaling transduction.

Because EDCs act via the same molecular mechanisms, they exhibit similar properties to hormones, which are distinct from those of classical toxic compounds. For example, like hormones, many EDCs act at low levels within the range of human exposure. Moreover, their effects depend on a series of variables such as the type of target tissue, the dose, the period of life at which exposure occurs, the sex and age of the individual, and other factors; as such, it is often difficult to predict the final outcome of exposure (34).

The developmental windows that are most vulnerable to EDC exposures largely determine the final phenotype of the exposed organism. EDCs act across the lifespan; however, exposure during pregnancy confers greater risk than during adulthood because they impact organogenesis, resulting in persistent effects throughout life (38). During pregnancy, the fetus is exposed to both persistent EDCs stored in the mother as well as very common nonpersistent EDCs. EDCs alter the expression of important genes during fetal development resulting in early morphological changes that may increase disease susceptibility. Similarly, the environment changes epigenetic marks that are transmitted from a cell to its daughter cell, a way of conferring signaling from events that occurred during development to the adult stage. There are numerous examples of morphological alterations produced by developmental EDC exposures, including developmental alterations in the mammary gland, prostate, and nervous system (33, 34) as well as disruptions in adipose tissue, liver, and the endocrine pancreas (27). Importantly, these changes are not restricted to embryogenesis, but also occur during adulthood, as evidenced by exposure during pregnancy being a risk factor for mothers as well as offspring (39).

Particularly concerning is evidence that EDCs not only affect the exposed individual and its progeny but may also affect subsequent generations that were not directly exposed. Such transgenerational effects on lipid metabolism and obesity have been demonstrated in animal models (40, 41). Although we await epidemiological results in humans, animal data indicate that the increased incidence of noninfectious diseases could be in part due to exposures of our great- and great-great-grandparents to certain EDCs such as DDT (1,1′-(2,2,2-trichloroethane-1,1-diyl)bis(4-chlorobenzene); CAS 50-29-3), whereas our current exposures could adversely impact our descendants.

The importance of gestation as a window of vulnerability does not detract from the importance of exposure during adulthood. Exposure of the adult population produces effects that are reversible in most cases; however, it is essential to account for them, especially in cases where continuous exposure occurs over long periods of time.

### Endocrine Disrupting Chemicals in Plastics

Recently, numerous reports have shown that plastic waste is accumulating in natural habitats. Micro- and nanoplastics have been found in marine and fresh waters (42, 43) as well as on land. Plastics not only pose a threat to wildlife (44) but also to human health (42, 45). The main sources of human exposure are thought to be food and drinking water. Plastics have been found in human feces, gastrointestinal tract, lungs, and placenta (46, 47). Two main aspects threatening wildlife and humans are their physical effects (related to particle size, shape, and concentration) and their chemical effects (48).

A number of chemicals are added to plastics during manufacturing. These so-called plasticizers give each plastic unique properties and include stabilizers, flame retardants, antioxidants, and antimicrobials among others (49, 50). Many of these chemicals behave as EDCs, disrupting endocrine signaling after migrating from plastics to humans and wildlife (42, 51, 52).

In this review, we focus on two widespread, nonpersistent chemical classes, bisphenols and phthalates. Per- and polyfluorinated alkyl substances (PFAS) are persistent pollutants commonly found in plastics as well. Evidence linking PFAS and other persistent pollutants with diabetes mellitus has been recently published in a comprehensive review by Hoyeck et al. (31), and we recommend it for those interested in the effect of PFASs on β-cells.

## BISPHENOLS

### Overview of Bisphenols

Bisphenols (BPs) are a group of chemicals with a chemical structure based on two hydroxyphenyl functional groups most often linked by a methylene bridge, although there are important exceptions such as bisphenol-S (BPS) (4,4′-sulfonyldiphenol; CAS: 80-09-1), bisphenol-P [4,4′-(1,4-phenylenediisopropylidene)bisphenol; CAS 2167-51-3], and bisphenol-M [4,4′-(1,3-phenylenediisopropylidene)bisphenol; CAS 13595-25-0]. The bisphenols best studied for endocrine-disrupting action are bisphenol-A (BPA) [(propane-2,2-diyl)diphenol; CAS: 80-05-7], BPS and bisphenol-F (BPF) (4,4′-methylenediphenol; CAS 620-92-8), analogues used as BPA alternatives, and tetrabromobisphenol-A (TBBPA) [4,4′-(propane-2,2-diyl)bis(2,6-dibromophenol; CAS 79-94-7], used as a flame retardant. In this review, we focus on BPA and its alternatives. These BPs are widely used during the production of various plastic materials, including food and beverage containers, toys, medical equipment, and baby bottles.

Herein, we focus on the effects of BPs on glucose homeostasis and pancreatic β-cell function and viability. Recent reviews have addressed diabetes, obesity, and nonalcoholic fatty liver disease (5, 27, 53, 54).

### Human Studies

In 2008, Lang et al. (55) published a cross-sectional study providing the first evidence associating elevated urinary BPA levels with T2D and other health effects. Since then, other cross-sectional studies have linked BPA exposure and T2D (56–59). Indeed, a systematic review and meta-analysis concluded that several EDCs, including BPA, were associated with the prevalence of T2D (60). Interestingly, at least two reports showed clear evidence of impaired glucose homeostasis, with higher urinary BPA levels associated with hyperinsulinemia and insulin resistance (61) and higher glycosylated hemoglobin (HbA1c) levels (62). In both studies, the associations were sex-dependent, with stronger effects in men. Alterations in insulin release have been demonstrated in humans following ingestion of a single dose of 50 µg/kg of BPA. These results identified a strong positive correlation between HbA1c and percent change in the insulinogenic index, an indicator of early-phase insulin release in young nonobese individuals. In contrast, older obese individuals exhibited a decrease in insulin and C-peptide levels following BPA ingestion (63). The prevalence of obesity is also associated with BPA exposure in children and adolescents (64), an effect also observed for BPA alternatives, such as BPS and BPF (65). BPS was associated with T2D whereas BPF was not (64).

Although cross-sectional studies are limited in the causal inference, especially when single biomarkers are used to describe complex metabolic traits, recent prospective studies using multiple exposure assessments have revealed both BPA and BPS to be positively associated with T2D, independent of traditional risk factors, such as caloric intake (66). Another prospective study demonstrates that BPA exposure was associated with decreased HOMA-B, indicating reduced β-cell function, and higher fasting plasma glucose levels before the development of diabetes in middle-aged and older women (67).

### Animal Studies

#### Adult exposure.

Evidence in animal models, mainly rodents, affirms the capacity of BPs to alter glucose homeostasis. These studies examine the impact of BPs on individuals during adulthood or the effects on offspring or mothers following BPs exposure during gestation. Most studies have examined BPA, a focus of this review; however, new data on other BPs have recently been published and are discussed briefly. Throughout this review, we will not give details of doses and duration of treatments in the text, yet interested readers can refer to the supplemental tables containing all these details (see Supplemental Tables S1–S4; https://doi.org/10.6084/m9.figshare.22709869).

In 2006, Alonso-Magdalena et al. (68) showed that adult male mice given a 4-day treatment with a low dose of BPA (100 µg/kg/day) developed insulin resistance and nonfasting hyperinsulinemia. Islets from treated animals exhibited increased insulin content and enhanced GSIS, effects potentially mediated by estrogen receptors, as BPA effects were not observed in mice treated with the pure antiestrogen ICI182,780 (ERα/ERβ antagonist).

**Figure 2.**
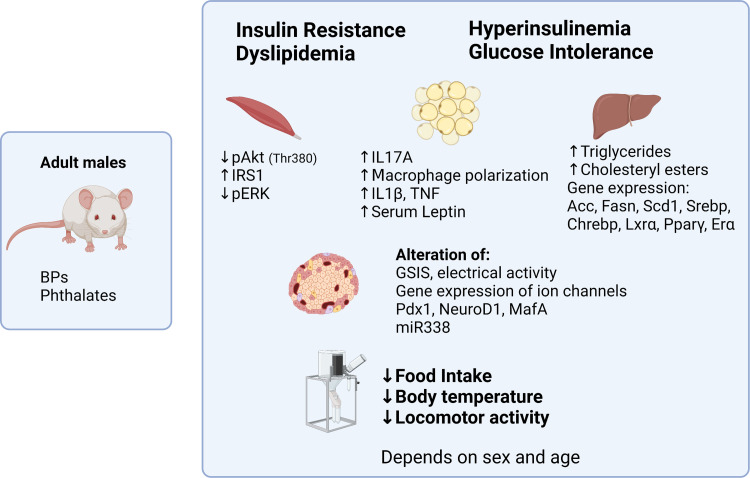
Metabolic phenotype of adult male mice treated with bisphenols (BPs) and phthalates. The studies summarized in this review give a general phenotype of insulin resistance and glucose intolerance along with hyperinsulinemia in the case of BPs and hypoinsulinemia in the case of phthalates. Bisphenol-A (BPA) modifies food intake, body temperature, and locomotor activity, although the results are scarce and should therefore be taken with caution. Both, BPs and phthalates, can induce dyslipidemia. Phenotypes have been better studied for BPA and the main end-points altered are specified under each organ in the figure. In the case of skeletal muscle, there are very few BPA studies, and the results are taken from Ref. 69. Studies of the BPA effect on adipocytes in adult rodents are not abundant either. In the figure, the results for adipocytes are from Ref. 24 and in the case of liver from Ref. 71. In general, the phenotype depends on age and sex, being more evident in males than in females, although more comparative studies between sexes should be carried out. ER, estrogen receptor; PPARγ, peroxisome proliferator-activated receptor gamma. Created with BioRender.com.

Other studies in adult male mice treated with low doses of BPA for one to several weeks reported induction of insulin resistance, glucose intolerance, and hyperinsulinemia in either the nonfasted (69) or fasted (70) state (Fig. 2). In both studies, ex vivo examination of islets revealed increased insulin content and enhanced GSIS as well as increased expression of Pdx-1 at mRNA and protein levels and increased levels of NeuroD1 mRNA without modifying Nkx6.1 or Mafa levels (70). These islets also had decreased expression of miR-338 (a microRNA that regulates Pdx-1), a finding replicated by in vitro exposure of isolated islets. Both in vivo and in vitro, BPA modifies the expression and function of Ca^2+^, Na^+^, and K^+^ channels in mouse pancreatic β-cells in an estrogen receptor β-dependent manner. This alters the electrical activity of the cell and insulin granule exocytosis, potentiating GSIS (72, 73). Animal models of higher BPA exposure showed increased body weight, altered lipid profiles (reduced HDL cholesterol), and shifts in the antioxidant status of the pancreas, suggesting that BPA exposure might induce hyperglycemia and its complications in adult male mice and rats by promoting oxidative stress (74, 75).

Importantly, GSIS alterations may be either the cause or consequence of the insulin resistance observed in BPA-treated mice. BPA has direct effects on pancreatic β-cells, increasing insulin content in isolated islets in an ERα-dependent manner (76). In vitro, the expression of miR-338 changed over time, showing a decrease up to 6 h of BPA exposure, but an increase from 12 to 48 h. This indicates that BPA modulates miR-338 levels, thereby controlling Pdx-1 expression (70). Unfortunately, neither study studied female mice nor examined sex differences in response to BPA.

These data indicate that β-cells could be a primary target of BPA, inducing postprandial insulin hypersecretion and causing insulin resistance. Critically, it is worth noting that BPA may also affect other tissues, including liver, skeletal muscle, and adipose, thereby altering insulin sensitivity (69, 77). For instance, adult male mice exposed to BPA acutely or for 2 wk exhibited suppressed hepatic glucokinase activity that may impair glucose sensing (78). Long-term exposure (8 mo) of male mice to BPA induced hyperglycemia, glucose intolerance, and hypercholesterolemia with an overexpression of key genes involved in hepatic cholesterol biosynthesis (79). In zebra fish, BPA can exacerbate existing metabolic stress induced by hyperglycemia and increase cellular senescence and increase apoptosis, both effects leading to aggravation of a T2D-like phenotype (80).

Collectively, data from adult BPA exposure models suggest that BPA simultaneously affects most organs regulating energy metabolism with consequential disruption of glucose homeostasis (Fig. 2).

#### Multigenerational effects.

**Figure 3.**
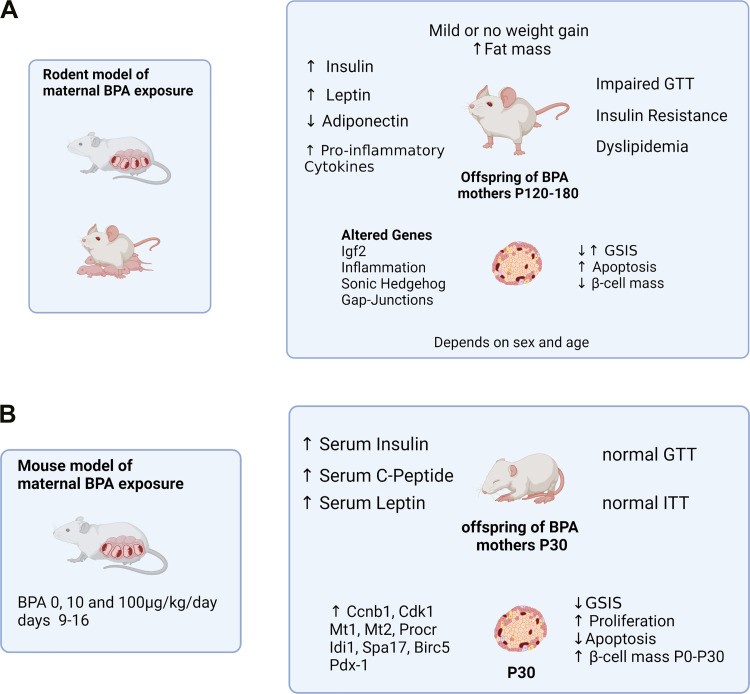
Metabolic phenotype of offspring from bisphenol-A (BPA)-treated mothers. *A*: the later-in-life phenotype of *P120*–*P180* has been best studied by many different groups in mice and rats. Common variables in most studies show increased fat mass with little or no weight gain along with insulin resistance, impaired glucose tolerance, dyslipidemia, and hyperinsulinemia. The β-cells of these animals exhibit altered gene expression and increased or decreased glucose-stimulated insulin secretion (GSIS) depending on the study, increased apoptosis, and decreased β-cell mass. This phenotype is more marked in males than in females and occurs as well in the second and third generation (87). *B*: early changes from *P0* to *P30* consist of hyperinsulinemia and hyperleptinemia without modification of insulin sensitivity or glucose tolerance. These changes are associated with increased β-cell mass from *day P0* and a large number of gene expression changes along with increased β-cell proliferation, decreased apoptosis, and increased β-cell mass at the early age of 1 mo (89). GTT, glucose tolerance test; ITT, insulin tolerance test. Created with BioRender.com.

**Figure 4.**
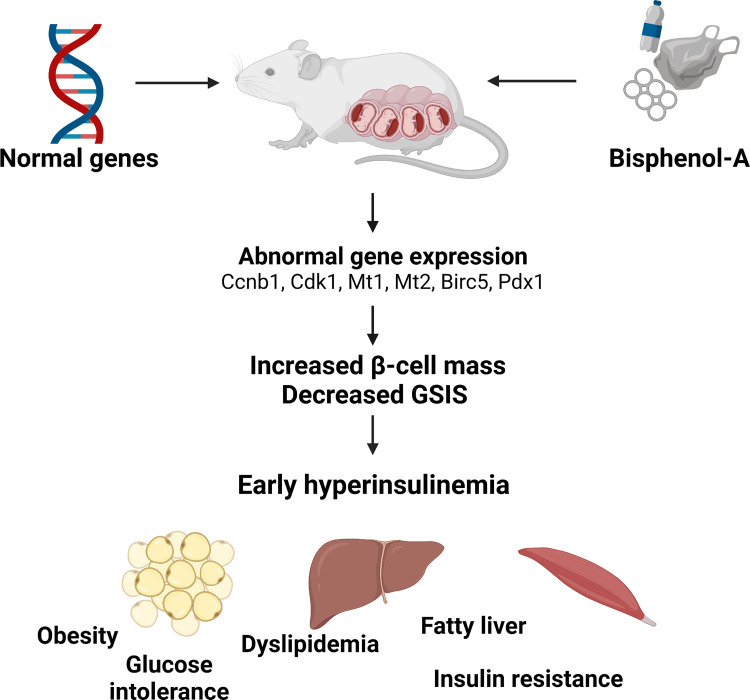
Hypothetical model of how the early phenotype induced by bisphenol-A (BPA) may contribute to metabolic alterations later in life. Bisphenol-A treatment of pregnant mice exacerbates insulin resistance in mothers (81) and can cross the placental barrier and produce effects in fetuses. As a consequence of these BPA actions, there is a modification of the expression of genes involved in cell division and function that should contribute to early hyperinsulinemia, increased β-cell mass and decreased glucose-stimulated insulin secretion (GSIS) (89). This early hyperinsulinemia should contribute to weight gain later in life, as well as glucose intolerance, dyslipidemima, insulin resistance, and fatty liver that have been demonstrated in adult male mice in many different studies. Created with BioRender.com.

Numerous investigations have shown that BPA exposure during fetal development alters glucose metabolism. In 2010, it was shown that BPA exposure as low as 10 µg/kg/day decreased insulin sensitivity, impaired glucose tolerance, and increased plasma concentrations of insulin, triglycerides, and leptin in dams. Male offspring exhibited glucose intolerance, insulin resistance, hyperinsulinemia, and hyperleptinemia, whereas female offspring were unaffected (81). Subsequently, altered glucose metabolism has been demonstrated in many works from different laboratories in both rats and mice (82–88) (Fig. 3*A*). In contrast, one study did not detect differences in glucose metabolism (90). Mechanistically, gestational BPA exposure may alter fetal physiology by perturbing metabolic pathways, including bile acid and tryptophan metabolism in the fetal liver (91) or hepatic retinoid signaling (92), that result in dysregulation of glucose metabolism and pancreatic function (92). Importantly, early changes in metabolic parameters of offspring exposed in utero to BPA include nonfasting hyperinsulinemia as an early marker followed by insulin resistance and/or glucose intolerance as well as islets with decreased insulin content and impaired GSIS ex vivo (89). These offspring exhibited greater β-cell area from the first day of life with evidence of decreased apoptosis and increased β-cell proliferation (89) (Fig. 3*B*). The increased β-cell mass and β-cell proliferation required ERβ as effects were absent in ERβ knockout mice (93). These early life actions are important because fetal life represents a critical period during which proper β-cell function and mass are established. In this case, it is possible that cell cycle-related regulation of gene expression programs greater β-cell mass from birth, as well as a reduced GSIS through increased expression of genes such as Mt1 and Mt2, which have recently been linked to β-cell function (Fig. 4). This higher β-cell mass is associated with hyperinsulinemia in ad libitum-fed animals. Notably, the mass that was increased at early ages decreases after 4 mo, and in the studies at later ages, it has been shown that mass is actually reduced in animals whose mothers were treated with BPA (87, 89). Therefore, in view of these results, it is possible that BPA behaves as an EDC acting on the estrogen receptor ERβ, altering gene expression in islets during development and producing early hyperinsulinemia and hyperleptinemia. This hyperinsulinemia appears to precede subsequent insulin resistance and weight gain (89, 93) (Fig. 4).

Critically, phenotypic differences exist across these studies, effects that may be partly dependent upon whether treatment was performed during gestation or during gestation and lactation; however, common features in most studies using rodents aged several months, include impaired glucose tolerance, insulin resistance, hyperinsulinemia, decreased serum adiponectin, dyslipidemia, and decreased β-cell mass, with modest or no weight gain (Fig. 3*A*). The phenotype is sex-dependent, being stronger in males, and age-dependent, starting around 4 mo of age, with effects generally exacerbated by high-fat feeding. Importantly, the effects of BPA in rodents have been validated in larger animal models, such as sheep, which exhibit adipocyte hypertrophy and increased inflammation, although, in contrast to rodents, sheep show increased insulin sensitivity (94). In Drosophila, BPA treatment increased glucose and trehalose levels, insulin resistance, oxidative stress, and apoptosis (95).

Generally, it is assumed that BPA effects on offspring arise from transplacental passage with direct impacts on developing fetal cells. However, metabolic alterations in the mother caused by BPA could also be effectors of the ultimate offspring phenotype. Most likely, the final phenotype depends on both parameters: direct effects of BPA on the fetus and BPA-induced alterations in maternal metabolism during pregnancy (81). This supposition, however, requires further study. Note that BPA treatment during pregnancy also changes GSIS and pancreatic β-cell mass in mothers, months after delivery. This suggests that BPA exposure during pregnancy is a risk factor for T2D not only for offspring, but also for mothers later in life (39).

Islets from male mice exposed to BPA in utero exhibit increased GSIS, similar to that of high-fat diet (HFD)-fed mice (85), reflecting an adaptation to insulin resistance due to being overweight. Islets from BPA-treated mice on a HFD exhibited reduced GSIS, below that of HFD-fed controls (85). Other studies have described impaired GSIS in islets up to two generations postexposure, F1 (offspring of treated dams) and F2 (grandchildren of treated dams). These islets exhibited mitochondrial dysfunction, and animals had reduced β-cell mass with greater β-cell death compared with controls (87). Alteration of multiple genes associated with mitochondrial function and inflammation were also reported (87). Interestingly, the decreased β-cell mass has been described up to the third generation (F3), implying that individuals not directly exposed to BPA exhibit effects that must be inherited (96). This same group also showed that while prolonged paternal postpubertal exposure does not adversely affect offspring glucose homeostasis, paternal developmental BPA exposure can impair glucose tolerance in female offspring, providing evidence that paternal exposure to EDCs pose a risk to metabolic health in their progeny (97). Recent results show that BPA exposure of pregnant females causes obesity in the F2 generation, but not in the F1 generation; this obesity phenotype is subsequently transmitted up to the F6 generation. This transmission of obesity correlates with epigenetic changes in sperm showing alterations in sites containing binding motifs for the CCCTC-binding factor (CTCF) of the Fto gene (98).

Multigenerational actions may involve epigenetic changes. There are no epigenetic studies examining BPA effects on the endocrine pancreas; however, such studies exist for obesity. Progeny of BPA-exposed pregnant Agouti mice became yellow and obese, with decreased DNA methylation of the agouti gene (99). Early-life exposure to the pesticide DDT, as well as relatively high doses of other EDCs, induced obesity in the unexposed F3 generation (100, 101). Despite these findings, the mechanisms by which epigenetic changes are transmitted across generations are largely unknown. Interestingly, Bruce Blumberg’s laboratory is beginning to uncover mechanisms of epigenetic inheritance. They propose that the EDC tributyltin (TBT; CAS 688-73-3) alters chromatin architecture and that this altered structure may have the ability to self-reconstruct in unexposed generations giving rise to epigenomic alterations (40, 102). This model is able to explain DNA methylation, noncoding RNAs, and histone modifications, the three major types of epigenomic modifications implicated as mediators of epigenetic memory across generations after ancestral EDC exposures (103).

Further studies are needed to understand the role of epigenetic modifications particularly in the endocrine pancreas.

#### Type 1 diabetes mellitus.

More data on EDC effects on type 1 diabetes (T1D) pathogenesis are urgently needed; however, data from the NOD mouse model indicate that in utero and lactational BPA exposure accelerates the spontaneous development of diabetes in this model of T1D. This acceleration appears to arise from immune system modulation early in life that promotes insulitis and diabetes development later in life (104, 105). Interestingly, this effect was enhanced when BPA and phthalate exposures were combined (106). Other studies using NOD mice revealed sex-dependent effects, with BPA accelerating T1D development in females but delayed it in males. In this model, BPA promoted inflammation and proinflammatory changes in the microbiome in females but with opposite effects in female offspring (107), and induced anti-inflammatory immune factors and decreased anti- and proinflammatory gut microbiota in males (108). Using STZ-induced diabetes as a T1D model, BPA altered the expression of genes regulating Ca^2+^ homeostasis, leading to endoplasmic reticulum stress and β-cell dysfunction (109); however, another report using this model showed that BPA restored the glucose intolerance and restored the insulin transcriptional regulators Pdx1, Mafa, and NeuroD1 (110). Finally, BPA effects were also investigated in a multiple, low-dose STZ-induced T1D model that revealed BPA to act as a potentially diabetogenic compound with immunomodulatory actions in the context of T cell immunity (111).

#### Bisphenols other than BPA.

Although human exposure to BPA has been clearly implicated in the development of diabetes, it is less clear that BPA analogs, such as BPS and BPF, can alter glucose homeostasis. However, several recent papers have shown that these BPA substitutes have similar effects to BPA. In male zebrafish, BPS significantly increased fasting blood glucose, decreased insulin levels, and altered the metabolism of the liver increasing gluconeogenesis and glycogenolysis (112). A study of gestational BPS exposure in rats revealed increased glucose tolerance, pancreatic β-cell proliferation, islets of smaller area, and β-cell size, suggesting estrogen-like effects of BPS, with males apparently more closely resembling females in their responses to a glucose challenge (113). When comparing different BPs, some studies suggest that BPS has strong obesogenic potential, whereas the BPF effects are subtler and potentially in the opposite direction (114). In other studies, BPA, BPF, and BPAF [4,4′-(1,1,1,3,3,3-hexafluoropropane-2,2-diyl)diphenol; CAS 1478-61-1] induced abnormal lipid and carbohydrate metabolism in zebrafish by inducing cellular changes in the gut. In addition, low doses of BPA and BPS disrupted the normal expression of pancreas-associated genes (Pdx-1, Foxa2, Ptfla, and Isl1) and the DNA methylation pattern of selected genes in early zebrafish development (115).

### Cellular Studies

In recent years, the need for urgent regulatory policies concerning EDCs has given rise to integrative strategies for identifying potential EDCs altering lipid and/or glucose homeostasis (5, 27). To this end, in vitro studies from human/mouse/rat-derived cell lines as well as primary cultures have become vitally important not only for their time and cost savings but also because of their power to predict EDC’s endocrine modes of action (116, 117). Herein, we summarize the main results of different cell studies that have evaluated the effect of BPs on β-cell function.

As previously mentioned, pancreatic β-cells control glucose homeostasis via GSIS as well as insulin biosynthesis and storage, all of which depend on electrical activity, Ca^2+^ signaling, mitochondrial activity, and expression of identity genes (72, 118, 119). In addition, endoplasmic reticulum and oxidative stress as well as disrupted mitochondrial activity are associated with β-cell death (120–122). Alterations in any of these variables by BPs or other EDCs could promote β-cell dysfunction, a hallmark of both T1D and T2D (123, 124).

#### Effects of bisphenols on β-cell function.

Several studies have assessed the impact of BPA on GSIS. Among them, only a few have also studied the effects of BPS and BPF. Since the effects of BPs change depending on treatment duration, it is crucial to differentiate between what would correspond to acute or short-term versus long-term treatment. We consider acute or short-term exposures as those between 0 and 4 h, and long-term exposures as those between 12 and 72 h.

Most groups have reported that BPs increase insulin secretion at basal and stimulatory glucose concentrations [hereafter referred to as basal insulin secretion (BIS) (2.6–5.6 mM glucose) or glucose-stimulated insulin secretion (GSIS) (6.7–20 mM glucose), respectively], following acute or short-term exposures and with doses of EDCs ranging from 1 nM to 100 μM, in primary mouse islets, INS-1E and β TC-6 cell lines (70, 125–128). In addition, it has been observed that 1 nM BPA blocked K_ATP_ channel activity following 3 to 7 min of BPA treatment while augmenting glucose-induced intracellular Ca^2+^ oscillations, a set of responses tightly associated with enhanced GSIS (127, 129, 130). K_ATP_ channel inhibition was also seen with BPS and BPF exposure for 10 min along with increased GSIS (126).

In contrast with these reports, two studies described no effects on GSIS following with 2-h BPA exposure in INS-1 832/13 or isolated Wistar rat islets (131, 132), although one reported an increase in BIS at 10 and 100 nM (132).

In summary, short-term treatments point to rapid BPs-induced insulinotropic effects, with the vast majority of the studies using environmentally relevant concentrations (133). Importantly, this action likely arises from rapid nongenomic responses leading to K_ATP_ inhibition and increased intracellular Ca^2+^ signaling (127, 129, 130).

Long-term exposures show more heterogeneous results. Regarding BPA, five studies reported increases in BIS, GSIS, or insulin content at concentrations within the nanomolar range (from 1 nM to 438 nM) after 24–48 h of treatment using MIN-6, β TC-6, and INS-1 cells as well as mouse and rat pancreatic islets (76, 118, 126, 131, 134–136). In the human EndoC-βH1 cell line, however, BPA induced no effects at 24 h, but increased GSIS at 72 h at doses from 10 nM to 1 μM (118, 137). Notably, some of these increases occurred without altering insulin content (126). In contrast, at similar or slightly higher BPA concentrations, five papers reported reduced GSIS or insulin content using the same cell types reporting long-term increases in GSIS (70, 125, 134, 136, 138). It is notable that some of these effects exhibited nonmonotonic dose responses, specifically with higher doses in the µM range decreasing GSIS, whereas lower doses in the picomolar and nanomolar range increased GSIS (70, 136). BPS and BPF increased GSIS at 1 nM and 1 μM after 48 h without altering insulin content in mouse pancreatic β-cells (126); however, BPS also produced decreased GSIS and insulin content at doses as low as 100 pM in MIN-6 and 1 nM in EndoC-βH1 after 24 h and 48 h of exposure, respectively; BPF showed no effects (118).

Several groups have more deeply studied the molecular mechanisms responsible for the complex actions of these chemicals on the pancreatic β-cell. As mentioned, insulin secretion and biosynthesis are largely dependent on electrical activity, maturation state, and mitochondrial activity, all of which rely on protein and gene expression as well as ion channel activity. BPA and BPS modify the expression of identity or function-related genes in rodents as well as in the EndoC-βH1 human cell line, sometimes with opposite effects. Downregulation or upregulation of these genes depends on the cell type and treatment duration. The most frequently altered genes are Snap-25 (exocytosis), Pdx1 (identity, maturation, and survival), and Hnf1a (differentiation) (70, 118, 136). In addition, reductions in voltage-gated Na^+^, K^+^, and Ca^2+^ ion channel expression have been observed with BPA, BPS, and BPF with nanomolar doses (1 nM for BPA/BPS, 100 nM and 1 µM for BPF) upon 48 h of exposure. Importantly, decreased gene expression occurs concomitantly with reduced Na^+^, K^+^, and Ca^2+^ currents, resulting in altered overall electrical activity, as indicated by increased burst duration, action potential amplitude after hyperpolarization, and area-under-the-curve (72, 118, 126).

BPs also affect mitochondrial ATP production, with 24–48 h of BPA exposure at 1 nM to 2 μM resulting in decreased ATP content, mRNA expression of mitochondrial function genes, and cytochrome c oxidase (COX) (a mitochondrial function marker) in mouse and rat islets or INS-1 and β TC-6 cell lines (70, 134–136, 138).

Overall, BPs have the capacity to alter β-cell gene expression, electrical activity, and mitochondrial function, all of which impact insulin biosynthesis and GSIS (Fig. 1*C*).

#### Bisphenols effects on β-cell viability and proliferation.

Another feature of BPs is their ability to affect β-cell viability that is directly related to dose and exposure duration such that higher doses or incubation times often led to an enhanced β-cell death (70, 118, 125, 135–142). The concentrations used across the different studies comprise low or environmentally relevant doses (pM to nM) and high doses (μM), in mouse/rat islets, mouse/rat cell lines, and human cell lines. With 24-h exposure, effects are observed in INS-1E and MIN-6 cells at picomolar concentrations (137), and in the nanomolar range for BPF in EndoC-βH1 cells (118). In contrast, one study did not find decreases in cell viability at nanomolar concentrations of BPA after 72 h of incubation of INS-1 cells (125).

Concomitant and likely related to apoptotic effects was the induction of endoplasmic reticulum and cellular stress markers (Fig. 1*C*). Briefly, BPA augmented Bax/Bcl-2 ratio, CHOP, Apaf-1 protein, cleaved caspases, cytosolic cytochrome C, NF-κβ, pERK_1/2_, and ceramides levels; it also altered the endoplasmic reticulum chaperones expression, including glucose-regulated protein (GRP)78, GRP94, and heat shock protein (HSP)70, in the nM-μM range upon 24–48 h of exposure (70, 128, 134, 135, 138, 140, 142, 143). Endoplasmic reticulum dysfunction and mitochondrial failure are tightly associated with each other since these two organelles communicate with each other via mitochondria-associated membranes, and have important roles in intracellular Ca^2+^ signaling, cell viability, mitochondrial network integrity, and lipid biosynthesis among other pathways (119, 144). Remarkably, BPs alter mitochondrial activity by means of loss of mitochondrial membrane potential, mitochondrial fragmentation [through decreased transcription factor A, mitochondrial (Tfam)], and increased expression of Ucp-2 and decreased expression of Ogdh (134, 136, 138). In addition, BPA increases reactive oxygen species (ROS) and antioxidant mitochondrial genes (135, 137, 139, 141). All of these mitochondrial disruptions were produced in the same dose and time range as those used to measure cell viability.

Misfolded human islet amyloid polypeptide (hiAPP) induces membrane permeabilization and ROS in β-pancreatic cells and is common in T2D. It has been reported that high doses of BPA (7.5 to 150 µM) accelerated hiAPP misfolding when INS-1 cells were incubated with hiAPP, and that BPA increased hiAPP-induced ROS production and cell apoptosis in a synergistic dose-dependent manner (139). Conversely, other BP analogues [BPAF, BPAP (4-[1-(4-hydroxyphenyl)-1-phenylethyl]phenol; CAS 1571-75-1], TBBPA, TCBPA (2,6-dichloro-4-[2-(3,5-dichloro-4-hydroxyphenyl)propan-2-yl]phenol; CAS 79-95-8) in doses ranging from 1 µM to 10 μM upon 24 h of exposure attenuated hiAPP aggregation and its membrane disruptive effects. In this case, none of the bisphenols (BPB (4-[2-(4-hydroxyphenyl)butan-2-yl]phenol; CAS 77-40-7), BPF, BPS, BPAF, TBBPA, TCBPA) affected cell viability under these specific conditions (145).

In contrast to BPA, little is known about the possible apoptotic effect of other BPs. In one study, low doses of BPS and BPF modified mitochondrial activity and induced apoptosis (118), whereas another showed no effect of these compounds (145).

Several studies point to BPs affecting β-cell proliferation. It has been observed that 10 µM BPS and BPF increase cell proliferation in BxPC3 (β-pancreatic tumor cell line) after 24 h (145). Boronat-Belda et al. (93) found increased cell proliferation of mouse islet β-pancreatic cells treated with 1, 10, and 100 nM BPA for 48 h. Of the three doses, 1 nM was the only one that increased neonatal mouse β-cell proliferation. In addition, 1 nM BPA and 10 µM BPS for 24 h also increased cell proliferation; however, higher doses of BPA or BPS (100 µM) negatively affected cell confluence and induced cytotoxicity in INS-1E cells (143). Negative effects on cell growth rate were also reported in β TC-6 cells after treatment with 2, 20, 100, and 200 nM BPA for 24 h, resulting in reduced cell number in G1, S, and G2/M phases and increased apoptosis in G0/G1 phase (135). This variety of effects on cell proliferation could also be explained by the opposing effects of ERα and ERβ, but further evidence would be needed to draw definitive conclusions regarding the in vitro effects of BPs on cell proliferation (146).

## PHTHALATES

### Overview of Phthalates

Phthalates are a group of chemicals widely used in a range of cosmetic, personal care, medical, and food packaging products (147). Phthalates are not covalently bound to plastics and therefore may leach out and act as EDCs based on their capacity to interact with several nuclear receptors. Some phthalates bind to estrogen receptors, making them weakly estrogenic, while other phthalates antagonize androgen receptor signaling, thus altering reproduction (148). Some high molecular weight phthalates antagonize thyroid receptors and affect metabolism (149). Several phthalates alter the function of different peroxisome proliferator-activated receptors (PPARs), receptors with major roles in lipid metabolism and energy homeostasis (150). Phthalates activate the aryl hydrocarbon receptor, which is essential for xenobiotic metabolism, and phthalates may also affect liver X receptors that are important regulators of cholesterol, fatty acid, and glucose homeostasis (151). Phthalates are classified into two groups based on their molecular weight. Low-molecular-weight phthalates (LMW, contain ester side-chain lengths of one to four carbons), include DMP (dimethyl benzene-1,2-dicarboxylate; CAS 131-11-3), DEP (diethyl benzene-1,2-dicarboxylate; CAS 84-66-2), DBP (dibutyl benzene-1,2-dicarboxylate; CAS 84-74-2), and DIBP (bis(2-methylpropyl) benzene-1,2-dicarboxylate; CAS 84-69-5), whereas high-molecular-weight phthalates (HMW, ester side-chain lengths of five or more carbons) include diethylhexyl phthalate (DEHP; bis(2-ethylhexyl) benzene-1,2-dicarboxylate; CAS 117-81-7), DOP (dioctyl benzene-1,2-dicarboxylate; CAS 117-84-0), and DINP (bis(7-methyloctyl) benzene-1,2-dicarboxylate; CAS 28553-12-0). Phthalates are used in personal care products and cosmetics as fragrance carriers and solvents; HMW phthalates are added to polyvinyl chloride (PVC) plastic to increase durability and flexibility in food processing and packaging materials, building materials, and medical devices (152). Ingestion is the main route of exposure in humans, with the following derivatives frequently found in urine: diethyl phthalate (DEP), DEHP, dibutyl phthalate (DBP), butylbenzyl phthalate (BBP; benzyl butyl benzene-1,2-dicarboxylate; CAS 85-68-7) and di-*i*-butyl phthalate (DiBP) (153). Among the HMW, DEHP is highly abundant in plastics, especially in food contact materials (154). DEHP together with BBP, DBP, DIBP, and DCHP (dicyclohexyl benzene-1,2-dicarboxylate; CAS 84-61-7) are added to increase flexibility, transparency, durability, and longevity, and they have been classified as substances of very high concern by Registration, Evaluation, Authorisation and Restrictions of Chemicals (REACH) regulation of the European Union (https://echa.europa.eu/hot-topics/phthalates). A distinguishing feature of these compounds is that they are rapidly metabolized and excreted in urine, confirming widespread exposure to these compounds (155, 156). It should be noted that some phthalate metabolites are biologically active as well (157). Phthalates have short half-lives in vivo; however, continuous and high human exposures to these compounds mandate examining their effects on health using long-term exposure frameworks.

### Human Studies

Urinary phthalates levels are associated with T2D risk in middle age women (158–160). Phthalate metabolites were positively associated with T2D in the general adult population in Korea (161), adult males (but not in females) residing in Shanghai (162), and in middle-aged women in the Nurses’ Health Study (NHS), NHSII cohorts (163), and SWAN study (160). In adolescents, urinary DEHP levels are associated with insulin resistance in the 2003–2008 NHANES (164), whereas urinary DINP in the 2009–2012 NHANES (165) and male adolescents with higher concentrations of MnBP (2-butoxycarbonylbenzoic acid; CAS number: 131-70-4) and MiBP [2-(2-methylpropoxycarbonyl)benzoic acid; CAS 30833-53-5] were associated with more components of the metabolic syndrome, including insulin resistance (166). A recent meta-analysis corroborates the positive association between phthalates and T2D (167), and the burden of diabetes in middle-age women due to phthalates has been estimated to be 20,500 annual new-onset cases in Europe with €607 million in associated costs (168), and 1,300 annual cases in the United States with $91.4 million in associated costs (169).

### Animal Studies

Rodent studies indicate that DEHP exposure causes reductions in pancreatic insulin content as well as changes in pancreatic β-cell morphology, leading to reductions in β-cell mass and alterations in blood glucose levels (170). Furthermore, exposure to phthalates increased body weight alone [DEHP, (171)] or in combination with a high fat diet, in this case inducing a fatty liver [BBP, (172)]. The effect of DEHP on glucose levels indicates that prolonged exposure in mice and rats increased fasting glucose levels, decreased serum insulin levels, and caused alterations in glucose tolerance (172–176) (Fig. 2). It should be noted that the effect of phthalates on glucose homeostasis decreases with time, likely due to induction of cytochrome P-450 and other enzymes capable of metabolizing them and accelerating their degradation (177). DEHP or diheptyl phthalate (DHP; diheptyl benzene-1,2-dicarboxylate; CAS 3648-21-3) administration also decreased plasma triglyceride levels (178, 179). Surprisingly, phthalate treatment in the NOD mouse model of T1D produced no effect on insulitis or T1D onset (106).

### Cellular Studies

The effects of phthalates on insulin secretion have been analyzed in mouse MIN6 cells, rat INS-1 cells, and the human cell lines EndoC-βH1 and 1.1B4. With acute exposure, high concentrations of DEHP (100 µM) applied for 2 h in INS-1 832/13 cells increased BIS, whereas GSIS was unaffected (132). In INS-1E cells, 100 μM MiBP, MnBP, or MEHP increased GSIS after 2 h of treatment (125). Given that there are few data about the acute effects of phthalates, more studies are required to better understand the rapid actions of these EDCs on β-cell secretory function and the potential mechanisms involved.

Studies using longer exposures (24–72 h) have generally documented inhibitory effects of phthalates [e.g., MBP (2-(butoxycarbonyl)benzoic acid; CAS 131-70-4), DEHP, DBP, and MEHP (2-(2-ethylhexoxycarbonyl)benzoic acid; CAS 4376-20-9] on BIS and GSIS after exposure to nanomolar concentrations (118, 180) and micromolar concentrations (118, 125, 180–183). At low nanomolar and micromolar concentrations, DEHP decreased GSIS in MIN6 cells and reduced insulin content at 1 µM (118). Nanomolar and micromolar concentrations of MEHP and MBP also reduced BIS and GSIS from INS-1 cells (180). This effect was accompanied by reduced expression of Ins-1, Ins-2, and Pdx-1, suggesting impaired insulin synthesis and reduced β-cell identity. In contrast, exposure of INS-1E cells to nanomolar concentrations of MiBP, MnBP, or MEHP did not significantly modify GSIS (125). DBP at micromolar ranges also inhibited both insulin synthesis and secretion from INS-1 cells and induced the downregulation of Glut-2 expression and increased Pdx-1 expression (181). The inhibitory effect of phthalates such as DEHP and/or DBP on β-cell function might be also related to altered mitochondrial function and increased oxidative and/or endoplasmic reticulum stress (181–183). However, given that most of these studies also observed reduced β-cell survival at similar concentrations, especially those in the micromolar range, it is likely that impaired β-cell secretion under these conditions may also be related to activation of proapoptotic pathways or cytotoxic effects. In any case, results from rodent cell lines generally indicate that phthalates negatively impact β-cell insulin secretion.

In human cell lines, long-term exposure to DEHP for 72 h did not affect insulin release in human EndoC-βH1 cells (118). However, exposure for 7 days led to reduced GSIS at 1–100 nM, whereas 1 μM increased secretion, suggesting nonmonotonic behavior. Moreover, DHEP decreased insulin content at 10–100 nM. Treatment of human 1.1B4 cells with nanomolar concentrations of MEP (2-ethoxycarbonylbenzoic acid; CAS 2306-33-4) for 24 and 72 h produced divergent effects on BIS and GSIS depending on doses and durations of exposure, revealing a high complexity of MEP actions on these cells (184). Therefore, although these few studies show that these phthalates also impair β-cell secretory function, further studies are required to clarify the complexity of phthalate actions in human cell lines.

#### Phthalates effects on β-cell viability.

The impact of phthalates on cell death and viability has been assayed in studies treating cells from 24 h to 7 days and include various cell models, including mouse MIN6 cells, rat INS-1 cells, human EndoC-βH1, and human 1.1B4 cells, as well as primary rat β-cells. Most studies reported decreased cell viability at nanomolar (180) and micromolar concentrations of phthalates such as DEHP, MEHP, MBP, and DBP (125, 181–183, 185–187). Al-Abdulla et al. (118) found that, while DEHP produced a limited impact on MIN6 cells at nanomolar concentrations, the viability decreased at micromolar doses. Similarly, no effect was found in INS-1 cells after exposure to nanomolar concentrations of MiBP, MnBP, and MEHP (125). At 30 μM, DEHP and DBP induced apoptosis in INS-1 cells, resulting in increased expression of apoptotic proteins such as Bax and caspases 3, 8, and 9 along with decreased expression of antiapoptotic proteins such as Bcl-2 (182). This treatment also led to increased ROS production (182). Indeed, a similar pattern of altered apoptosis-related protein expression augmented ROS production or oxidant levels and disrupted antioxidant defenses has been reported in several studies examining INS-1 cell exposure to phthalates, including DEHP, DBP, MEHP, and MBP (181–183, 187). Thus, oxidative stress might be a key process mediating the actions of phthalates on β-cell death and viability. It has also been reported that the detrimental effects of DBP and DEHP on INS-1 cell survival might be related to impaired PI3K/AKT signaling (182) as well as activation of endoplasmic reticulum stress (183). In primary rat β-cells, DBP-induced decreases in cell viability were associated with augmented STAT1 phosphorylation and downregulation of the transcription factor FoxM1 (186). Furthermore, MEHP increased lysosomal membrane permeability and cathepsin B release, leading to pyroptosis (inflammatory programmed cell death) and also affected autophagy in INS-1 cells (185). Thus, several mechanisms and signaling pathways appear to be involved in the deleterious effects of phthalates on β-cell survival in animal cell models.

In contrast to the aforementioned results in rodents, MEP had no impact on cell viability in human 1.1B4 cells at nanomolar concentrations (184). Indeed, MEP augmented cell proliferation after 72 h of exposure. In the case of human EndoC-βH1 cells, Al-Abdulla et al. (118) reported modest DEHP effects on cell viability at 1–100 nM and 1 μM. Therefore, although more studies are required, phthalates modestly affect human β-cell survival, at nanomolar levels.

## EFFECTS ON OTHER ISLETS OF LANGERHANS CELL TYPES

Unfortunately, the effect of endocrine disruptors on non-β islet cells is grossly understudied, with only a few publications on the glucagon-secreting α-cell. The α-cell is specialized to produce and secrete glucagon when plasma glucose levels are low, representing the first line of defense against hypoglycemia. Like other islet cell types, they behave plastically during periods when α-cell mass and function are needed, such as pregnancy, when estrogens and other hormones likely play a role (188). The effect of bisphenols and phthalates in α-cell viability and function has been studied mainly in the α-TC1-9 mouse cell line. At pico- and nanomolar range concentrations, BPA decreases αTC1-9 cell viability (189, 190) in a pathway that involves ROS production and, likely, estrogen receptors since these effects are abolished by the pure antiestrogen ICI182,780 (190). Other BPs such as BPS and BPF also decreased αTC1-9 cell viability (189). There are few studies using animal models; however, rats treated with 4.5 µg/L of BPA in drinking water exhibited increased α-cell apoptosis (75).

BPA was shown to acutely decrease low glucose-induced Ca^2+^ signaling in α-cells within intact islets of Langerhans (191). Impacts on low glucose-induced glucagon secretion, however, differ across studies and experimental conditions. BPA did not modify glucagon release after 48 h of incubation (190); however, secretion was decreased with 10 nM exposure for 24 h (189). Other BPs such as BPF and BPS decreased glucagon secretion as well as the expression of several genes involved in α-cell identity and secretion, including Foxo 1 (189), which is known to play an important role in α-cell function (192). Interestingly, at concentrations of 100 nM and 1 µM, the phthalate DEHP also decreased glucagon secretion (189). Studies of phthalate actions in animal models are scarce; however, treatment of zebrafish embryos with MEHP reduced α-cell area (193). These results reveal that glucagon-releasing α-cells may represent an important but underappreciated target of EDCs. It would be interesting to study the possibility that these EDCs alter the paracrine effects among, α, β, and the rest of cells of the islet of Langerhans. Therefore, more information on the effects of EDCs on the non-β-cells of the islet is urgently needed to better understand the role of these chemicals in modulating glucose homeostasis.

## SUMMARY AND CONCLUSIONS

Globally, the prevalence of diabetes mellitus is increasing rapidly with ∼10% of adults, or 537 million people, currently living with the disease and low- and middle-income countries increasingly affected. Furthermore, another 541 million adults have glucose intolerance, placing them at high risk for type 2 diabetes (https://diabetesatlas.org/). In this review, we have summarized a significant number of cross-sectional epidemiological studies as well as meta-analyses that support the role of exposure to BPs and phthalates in increasing the prevalence of diabetes among adults. Newer longitudinal studies have confirmed a role for BPA in altering glucose homeostasis and promoting the development of T2D. Few data are available regarding the possible role of other BPs in dysglycemia.

Importantly, T2D prevalence in children and adolescents is also rising in many countries due to increasing rates of overweight and obesity. While reliable data for this group are scarce, T2D is expected to become a serious public health problem in younger populations (https://www.idf.org/61-about/550-diabetes-in-children-and-adolescents.html). Epidemiological studies associate T2D and overweight/obesity with exposure to BPs and phthalates in children and adolescents, pointing to a possible role in the increased prevalence of T2D at younger ages.

Animal studies support the epidemiological evidence and illuminate the importance of dose, timing, age, and sex in determining EDC-dependent metabolic phenotypes. Exposure to BPA during adulthood decreases insulin sensitivity in most cases and produces hyperinsulinemia or hypoinsulinemia depending on the timing of exposure. These animals exhibited alterations in serum concentrations of other hormones, including leptin and adiponectin, as well as dyslipidemia. The phenotype may be more severe in males than in females, although most studies did not seriously consider sex differences and were only performed in males. There is a clear need to study the phenotype in both sexes. Reversibility has not been systematically studied, and although it is usually assumed in cases of adult EDC exposure as with other hormones (194), whether this is true is unknown.

Exposure during pregnancy, or during pregnancy and lactation, clearly predisposes the offspring to impaired glucose homeostasis. In most studies, around six months of age, males of BPA-treated mothers develop insulin resistance, hyperinsulinemia, hyperleptinemia, decreased adiponectin, dyslipidemia, and decreased β-cell mass. Not all studies included females, but those that did found no metabolic phenotype. Weight gain did not occur in most studies or occurred inconsistently across doses; thus the metabolic disruption is not directly dependent on weight gain. It should be noted that most studies did not include an analysis of fat mass. When we studied animals exposed during gestation to BPA during the first month of life, we found that on the first day of life they had greater β-cell area, and at day 30 mice had nonfasting hyperinsulinemia without alterations in glucose tolerance or insulin sensitivity. It is interesting to consider that hyperinsulinemia could be an early change that drives the altered metabolic phenotype later in life, namely insulin resistance (89, 195) and weight gain (196, 197) (Fig. 4).

Cellular studies clearly suggest that β-cells are an important primary target for BPs and phthalates. Treatment with both chemicals alters GSIS as well as β-cell viability and cell proliferation in some studies. Although the mechanisms are incompletely understood, they include altered gene expression and ion channel function, which modifies electrical activity and calcium signaling. Both classes of EDCs alter the expression of genes involved in β-cell function and differentiation, which play an important role in maintaining functional pancreatic β-cell mass, especially in response to nutritional stress (22). It is plausible that disruption of pathways leading to adaptation by BPs and/or phthalates accelerates the onset of T2D (Fig. 1). Although less studied, the effect of BPs and phthalates on the glucagon-producing pancreatic α-cell, namely a decrease in glucagon secretion, probably contributes to an impaired glucose homeostasis.

In conclusion, the data summarized in this review indicate that BPs and phthalates are associated with diabetes risk. Results for these two classes of nonpersistent pollutants along with those recently reviewed for persistent organic pollutants (31) show that β-cells are primary targets of EDCs. There is a high interest in the European Union in developing new tests to identify EDCs that may lead to obesity and diabetes. The work being developed within the Horizon 2020 European Union funded projects, GOLIATH, OBERON, and EDCMET (116, 117, 198), should accelerate the development of screening protocols using human β-cells (118, 137) to identify EDCs with diabetogenic action. These findings also support global efforts to negotiate a legally binding treaty (45) that would reduce the production and consumption of plastics and toxic chemicals used in plastic materials such as phthalates and bisphenols.

## SUPPLEMENTAL DATA

10.6084/m9.figshare.22709869 Supplemental Tables S1–S4: https://doi.org/10.6084/m9.figshare.22709869.

## GRANTS

The author’s laboratories are supported by Ministerio de Ciencia e Innovación, Agencia Estatal de Investigación (AEI), and Fondo Europeo de Desarrollo Regional (FEDER) Grant PID2020-117294RB-I00 (to A.N. and J.M.-P.); Generalitat Valenciana PROMETEO II/2020/006 (to A.N.); and European Union’s Horizon 2020 research and innovation program under Grant Agreement GOLIATH No. 825489 (to A.N.). This work was supported by National Institute of Environmental Health Sciences Grants R01 ES028879 and P30 ES027792 (to R.M.S.).

## DISCLOSURES

No conflicts of interest, financial or otherwise, are declared by the authors.

## AUTHOR CONTRIBUTIONS

J.M.-P. and A.N. prepared figures; J.M.-P., R.S.-N., R.M.M.-G., E.F., I.Q., R.M.S., L.T., and A.N. drafted manuscript; J.M.-P., R.S.-., R.M.M.-G., E.F., I.Q., R.M.S., L.T., and A.N. edited and revised manuscript; J.M.-P., R.S.-N., R.M.M.-G., E.F., I.Q., R.M.S., L.T., and A.N. approved final version of manuscript.
